# A network pharmacology-based approach to analyse potential targets of traditional herbal formulas: An example of Yu Ping Feng decoction

**DOI:** 10.1038/s41598-018-29764-1

**Published:** 2018-07-30

**Authors:** Huali Zuo, Qianru Zhang, Shibing Su, Qilong Chen, Fengqing Yang, Yuanjia Hu

**Affiliations:** 1 State Key Laboratory of Quality Research in Chinese Medicine, Institute of Chinese Medical Sciences, University of Macau, Macau SAR, China; 2 School of Pharmacy, Zunyi Medical University, Guizhou, China; 3 Research Center for Traditional Chinese Medicine Complexity System, Shanghai University of Traditional Chinese Medicine, Shanghai, China; 4 School of Chemistry and Chemical Engineering, Chongqing University, Chongqing, China

## Abstract

Herbal formulas from traditional Chinese medicines (TCMs) have been extensively used in clinics as effective therapies, but it is still a great challenge to demonstrate the scientific basis for their therapeutic effects at the level of molecular biology. By taking a classic herbal formula (Yu Ping Feng decoction, YPF) as an example, this study developed a novel network pharmacology based method to identify its potential therapeutic targets. First, this study constructed a “targets–(pathways)–targets” (TPT) network in which targets of YPF were connected by relevant pathways; then, this network was decomposed into separate modules with strong internal connections; lastly, the propensity of each module toward different diseases was assessed by a contribution score. On the basis of a significant association between network modules and therapeutic diseases validated by chi-square test (*p*-value < 0.001), this study identified the network module with the strongest propensity toward therapeutic diseases of YPF. Further, the targets with the highest centrality in this module are recommended as YPF’s potential therapeutic targets. By integrating the complicated “multi-targets–multi-pathways–multi-diseases” relationship of herbal formulas, the method shows promise for identifying its potential therapeutic targets, which could contribute to the modern scientific illustration of TCMs’ traditional clinical applications.

## Introduction

Understanding the scientific basis and therapeutic mechanisms of herbal formulas from traditional Chinese medicines (TCMs) is essential since they play a vital role in complementary and alternative therapies in clinical practice to cure diseases or restore the whole-body balance; additionally, they provide a rich source for drug discovery. Owing to the complexity of the constituents in TCMs, reductionism, which individually considers each ingredient, cannot adequately explain the effects generated by an entire formula, and therefore faces several challenges comprehending and illustrating the mechanisms of herbal formulas. With the rapid development of bioinformatics, a combined attempt based on system/network biology and polypharmacology has created a new paradigm called network pharmacology (NP).

In recent years, NP has been proposed as a promising approach for understanding herbal formulas^1,2^ and predicting potential new drugs or targets^3–5^. To some extent, NP-based research on the underlying therapeutic mechanisms of herbal formulas has been well-accepted^6,7^ and accounts for the strong compatibility of different scales of systems. A distinct advantage of network analysis is that it can excavate underlying information from multilevel interactions, especially when dealing with big data.

Yu Ping Feng (YPF) decoction, a classic herbal formula, has been used in clinics to treat various diseases, such as respiratory tract infection^8,9^, nosocomial pneumonia^10^, chronic bronchitis^11^, and perennial allergic rhinitis^12,13^. As a safe and effective formula, it has been reported to relieve idiopathic sweating and augment appetite in end-stage cancer patients^14^, prevent viral infections, such as severe acute respiratory syndrome^15,16^, and have immunoregulatory or beneficial effects in asthma^17,18^, hepatic-fibrosis^19^, pulmonary-fibrosis^20–22^, and dermatitis models^23^. Since explanations for the clinical application of YPF decoction have not yet been illustrated by the conventional method (such as the *in vitro* activity assays of selected compounds) due to difficulties in the experimental design of complex systems, its underlying mechanism remains to be explored.

**Figure 1:**
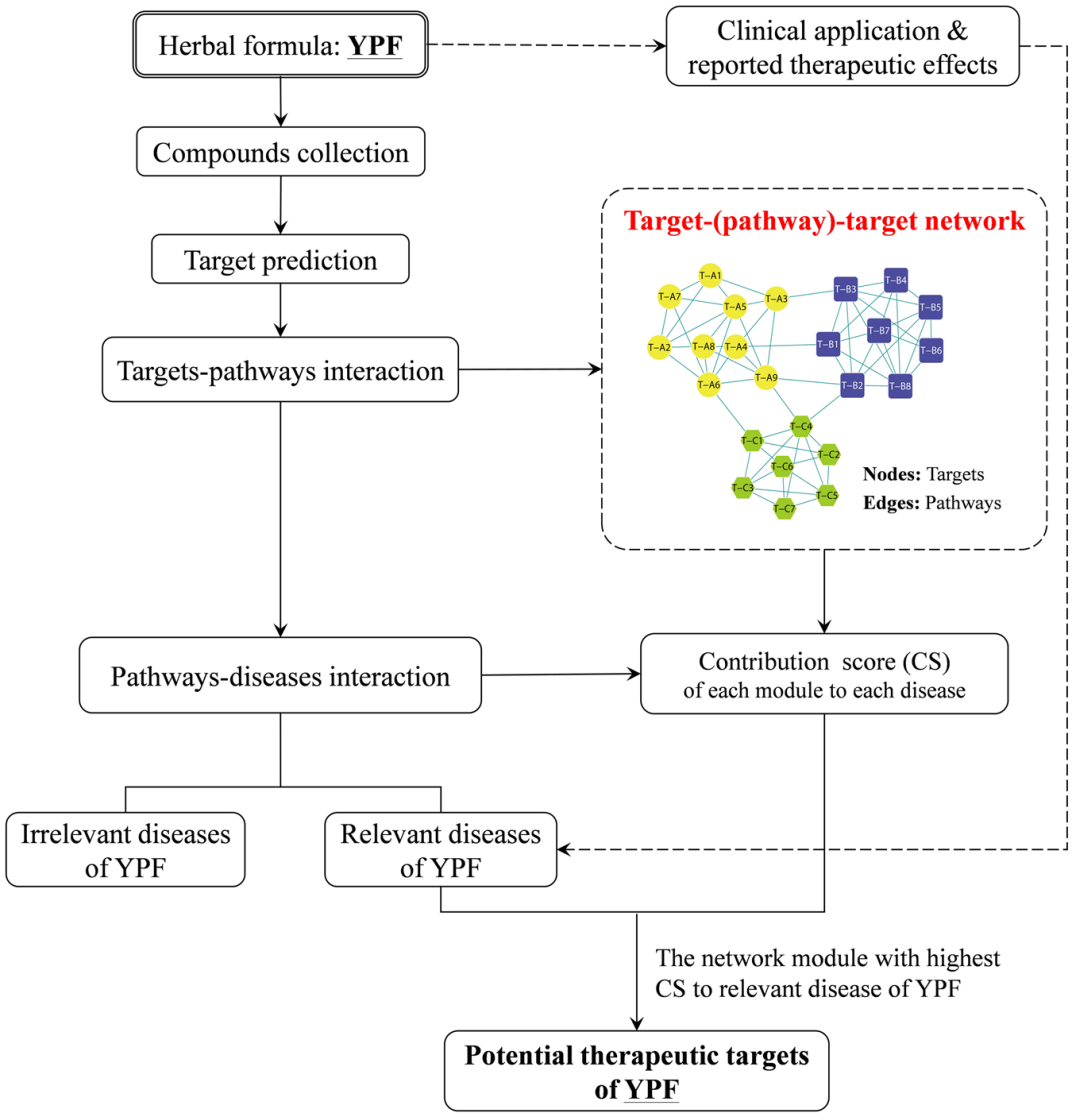
Workflow of the novel NP-based method.

Thus, this study aimed to develop an NP-based method for identifying potential therapeutic targets of traditional herbal formulas by taking YPF decoction as an example. This method gathered relevant bioinformatics information about YPF decoction to identify its underlying therapeutic targets and shed light on the NP-based mechanism of herbal formulas’ traditional clinical applications. The workflow of the NP-based approach and its application in YPF decoction is shown in below (Fig. 1).

In this study, first, the putative targets of compounds from YPF decoction were enriched to pathways to create the targets–pathways interaction, and the diseases related to these pathways were collected to create the pathways–diseases interactions. Next, the targets–pathways interactions were used to generate a “targets–(pathways)–targets” (TPT) network with partitioned modules (clusters), in the TPT network, targets act as nodes, and they are connected by relevant pathways (edges). Based on the TPT network, we hypothesised that if a group of protein targets were gathered to form a module naturally, they might share a functional similarity. Herein, a contribution score (CS) was calculated to evaluate the contribution of each module to different diseases based on the network modules’ partition and pathways–diseases interactions. Further, the module with the highest CS to relevant diseases of YPF as well as the notable targets within the module were identified to explore potential therapeutic targets of YPF. The detailed procedures are more fully described in the methods section.

## Results and Discussion

### The targets–pathways–diseases interaction profile of YPF decoction

YPF decoction consists of three herbs and a total of 352 compounds, which were collected with PubChem CID and they belonged to flavonoids, saponins, saccharides, and volatile components (including phenylpropanoids, monoterpenes, sesquiterpenes, diterpenes, and triterpenes). The rapid development of bioinformatics and computational sciences has provided a wide range of approaches for targets prediction of small molecules^24^. In this study, the putative targets of compounds were identified based on the similarity ensemble approach (SEA: http://sea.bkslab.org/)^25^. The potential targets of 250 compounds were successfully predicted by the SEA, with a total of 968 protein targets. (A worth-mentioning issue is about the high amount of 968 potential “targets” of YPF decoction, especially in terms of the relative small target pool of SEA. We tried to narrow the scope of potential “targets” of YPF decoction by setting up a pre-enrichment filter based on Tc threshold value. In this way, the number of “targets” for enrichment analysis sharply decreases from 968 to 304. However, the results of the most-important potential therapeutic targets and potential active compounds of YPF have no obvious changes. Thus, we still chose the post-enrichment filtering strategy, considering the advantage of this strategy with more informative reference to alternative targets. Throughout this paper, the term “targets” refers to the putative proteins to which the ingredients from YPF decoction directly bind, according to SEA predictions; however, it is not known whether targets are responsible for the therapeutic effect of YPF decoction. The term “therapeutic targets” refers to partial proteins from targets that have been identified to be responsible for the therapeutic effect of YPF decoction by our novel NP-based method.) Then, the target–pathway interactions were generated to elucidate the biological pathways that YPF decoction might impact; moreover, the overrepresented KEGG pathways of the predicted 968 protein targets of YPF were identified from enrichment analysis. A total of 132 highly related pathways with 549 relevant protein targets were obtained. Please refer to supplementary dataset for more detailed information about the compounds (Supplementary Table S1), putative targets (Supplementary Table S2) and relevant pathways (Supplementary Table S3).

To understand what role the enriched pathways might play from a therapeutic angle, the Kyoto Encyclopedia of Genes and Genomes^26^ (KEGG; http://www.genome.jp/kegg/pathway.html) was used to identify the diseases with which each pathway might be involved. Since each pathway might participate in various bioprocesses *in vivo*, it is also possible that each pathway could be relevant to multi-diseases, and that each disease might be contributed to by multi-pathways. Next, a total of 43 diseases that the 132 pathways might impact were retrieved from the KEGG database to create the pathways–diseases interaction. Finally, the targets–pathways–diseases interaction profile of YPF decoction was generated. (Throughout this paper, the term “diseases” refers to the disease category of the diseases relevant to the pathways. Please refer to Supplementary Table S4 for detailed information about the pathways-diseases relationship.)

### Targets–(pathways) –targets (TPT) network and module identification

Network modules (clusters or communities) refer to subnetworks whose nodes are more strongly connected to one another than to the rest of the network. That is to say, modules are sets of highly interconnected nodes. The identification of these modules is of crucial importance as they might help to uncover the hidden structural information within a network. Network modules have been utilised in NP-based TCM studies to uncover the combinations rule of herbal medicines^27^, chemical modules with similar structures^28^, and proteins with similar functions^29–31^. Especially in the context of TCMs as a typical complex system of multi-components and multi-targets, network modularity based of the Law of Like Attracts Like was considered to be a powerful way to sketch the complexity of TCMs.

**Figure 2:**
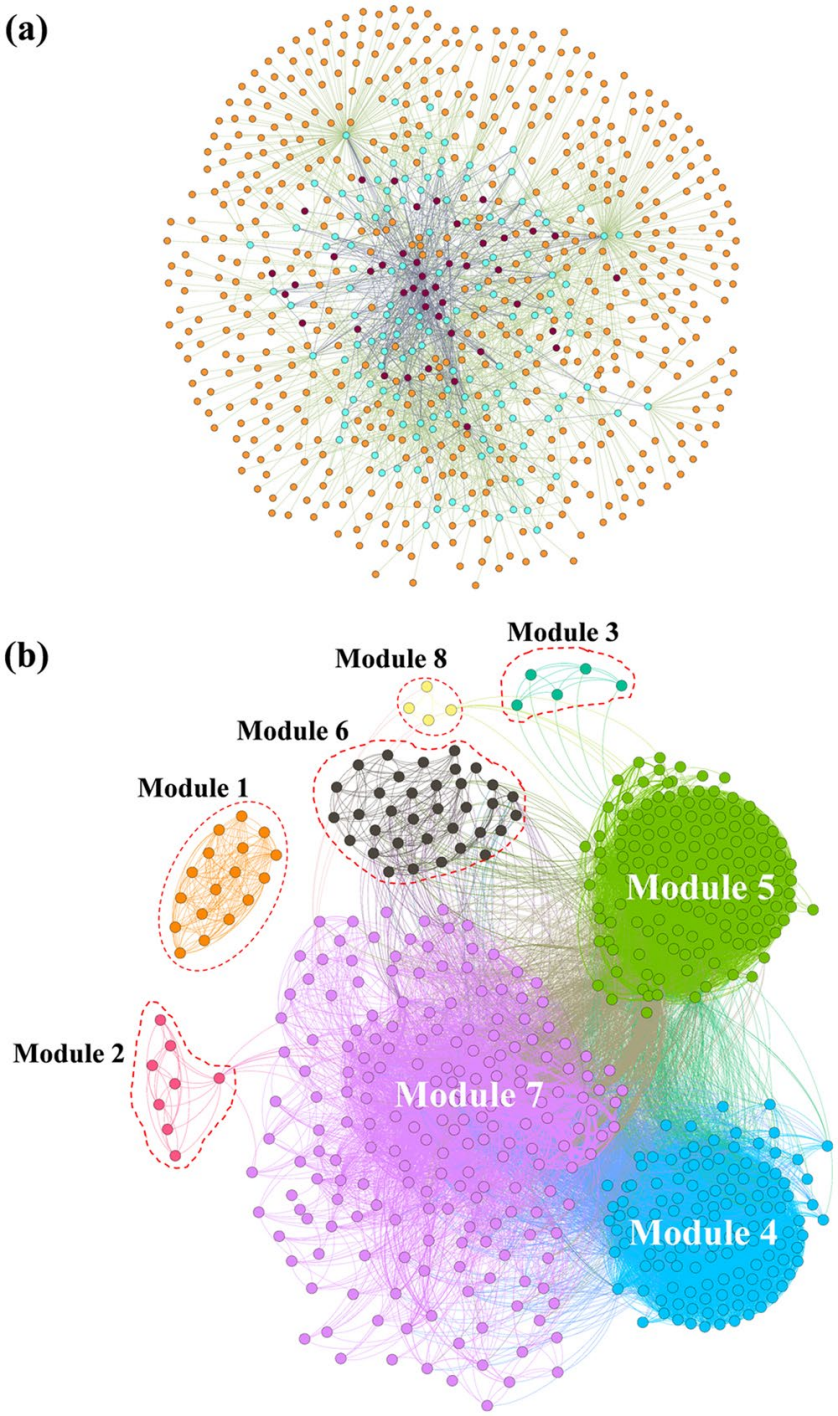
(**a**) Targets–pathways–diseases network, and (**b**) Targets–(pathways)–targets network with modularity partition. Nodes in (**a**) represent targets of YPF decoction (orange), relevant pathways (light blue), and diseases (deep purple), while edges represent the interaction of targets–pathways–diseases. Nodes in (**b**) represent the targets of YPF decoction, while edges refer to relevant pathways of these targets. If two nodes are connected by an edge, this indicates that they have been enriched to participate in at least one of the same pathways. Eight modules (modules 1–8) presented in different colours were detected and partitioned by the Louvain algorithm incorporated in the Gephi software.

The complicated targets–pathways–diseases interaction of YPF in Fig. 2(a) showed a preliminary NP-based bioinformatics profile of YPF decoction, by employing the most frequently used network analysis method in research on traditional remedies for therapeutic targets’ prediction. The nodes represent putative targets of YPF (orange), relevant pathways (light blue), and diseases (deep purple). From Fig. 2(a), it is difficult to determine which targets might be most relevant to the therapeutic effect that YPF exerts; therefore, the novel TPT network was established to realise the recognition of therapeutic-based targets of YPF decoction, as shown in Fig. 2(b). Eight modules (modules 1–8) were detected and divided by the Louvain algorithm incorporated in the Gephi software at a resolution of 1.0^32,33^. According to graph theory, the TPT network shown in Fig. 2(b) suggests some significant structural information, especially when considering the relatively separated modules with dense internal connections. Then, the inner interactions (edges representing the common pathways between every two nodes) of each target module were further investigated.

The targets in module 1, module 2, and module 3 were connected by the “proteasome” (hsa03050), “lysine degradation” (hsa00310), and “aminoacyl-tRNA biosynthesis” (hsa00970) pathways, respectively; the inner connection of the targets in module 6 was “protein digestion and absorption” (hsa04974) and in module 8, it was the “insulin signaling pathway” (hsa04910).

Three major modules (module 4, module 5 and module 7) could be easily identified from the network in Fig. 2(b). In addition, the inner connection of the targets in module 4 was the “neuroactive ligand-receptor interaction” (hsa04080) pathway. The connections between targets in module 5 were almost metabolism-related pathways, and the pathways in module 7 were pathways that immune-related, which was the most complicated. The results are shown in Table 1. From the results, we inferred that the target modules in the TPT network might represent the module shares’ different functional propensities. Thus, a contribution score (*CS*) was calculated from the newly established contribution-scoring algorithm to assess the relationship between each module and relevant diseases in the following study by integrating the modularity partition of the TPT network, target–pathway interactions, and pathway–disease interactions. The contribution-scoring algorithm is presented in the Method section in detail.

**Table 1:** The inner interactions (edges) of each target module.

Module	Pathway KEGG	Pathway description
Module 1	hsa03050	Proteasome
Module 2	hsa00310	Lysine degradation
Module 3	hsa00970	Aminoacyl-tRNA biosynthesis
Module 4	hsa04080	Neuroactive ligand-receptor interaction
Module 5	hsa00790	Folate biosynthesis
	hsa00330	Arginine and proline metabolism
	hsa00670	One carbon pool by folate
	hsa01100	Metabolic pathways
	hsa00270	Cysteine and methionine metabolism
	hsa00531	Glycosaminoglycan degradation
	hsa00600	Sphingolipid metabolism
	hsa00590	Arachidonic acid metabolism
	hsa00591	Linoleic acid metabolism
	hsa00140	Steroid hormone biosynthesis
	hsa00982	Drug metabolism - cytochrome P450
	hsa04913	Ovarian steroidogenesis
	hsa00980	Metabolism of xenobiotics by cytochrome P450
	hsa00350	Tyrosine metabolism
	hsa00592	alpha-Linolenic acid metabolism
	hsa00565	Ether lipid metabolism
	hsa00480	Glutathione metabolism
	hsa00830	Retinol metabolism
	hsa00380	Tryptophan metabolism
	hsa00360	Phenylalanine metabolism
	hsa00010	Glycolysis/Gluconeogenesis
	hsa00260	Glycine, serine and threonine metabolism
Module 6	hsa04974	Protein digestion and absorption
Module 7	hsa05169	Epstein-Barr virus infection
	hsa05100	Bacterial invasion of epithelial cells
	hsa04520	Adherens junction
	hsa05120	Epithelial cell signaling in Helicobacter pylori infection
	hsa05203	Viral carcinogenesis
	hsa04722	Neurotrophin signaling pathway
	hsa04670	Leukocyte transendothelial migration
	hsa04920	Adipocytokine signaling pathway
	hsa04144	Endocytosis
	hsa04062	Chemokine signaling pathway
	hsa05205	Proteoglycans in cancer
	hsa05134	Legionellosis
	hsa04210	Apoptosis
	hsa05161	Hepatitis B
	hsa05222	Small cell lung cancer
	hsa04115	p53 signaling pathway
	hsa05145	Toxoplasmosis
	hsa04330	Notch signaling pathway
	hsa04621	NOD-like receptor signaling pathway
	hsa05215	Prostate cancer
	hsa04064	NF-kappa B signaling pathway
	hsa05223	Non-small cell lung cancer
	hsa05416	Viral myocarditis
	hsa04612	Antigen processing and presentation
	hsa04620	Toll-like receptor signaling pathway
	hsa05133	Pertussis
Module 7	hsa05216	Thyroid cancer
	hsa03320	PPAR signaling pathway
	hsa05221	Acute myeloid leukemia
	hsa04660	T cell receptor signaling pathway
	hsa05212	Pancreatic cancer
	hsa05131	Shigellosis
	hsa04110	Cell cycle
	hsa05130	Pathogenic Escherichia coli infection
	hsa04914	Progesterone-mediated oocyte maturation
	hsa03410	Base excision repair
	hsa04012	ErbB signaling pathway
	hsa04150	mTOR signaling pathway
	hsa05321	Inflammatory bowel disease (IBD)
	hsa05211	Renal cell carcinoma
Module 8	hsa04910	Insulin signaling pathway

### Contribution score (CS) of modules toward diseases

**Figure 3:**
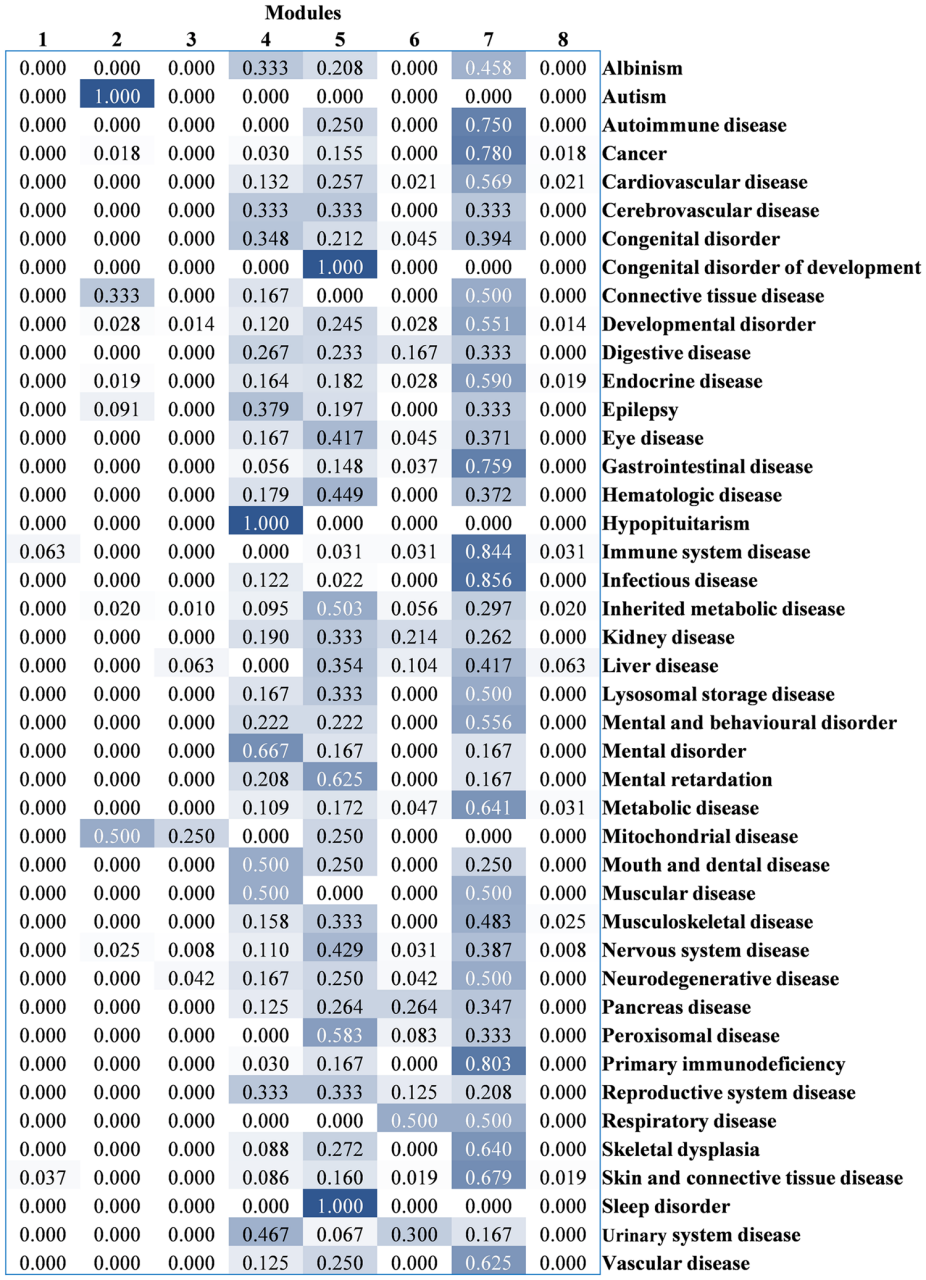
The contribution scores (CS) of targets in each module to different diseases. The deep blue and white refer to the highest to lowest CS, respectively.

Following the partition of the modules, the CS was calculated to evaluate the contribution of each module to each disease by the contribution scoring algorithm, the results of which are shown in a heat map (Fig. 3); the modules are listed in a row and the diseases in a column. The gradually changing colours, from deep blue to white, represent the highest to the lowest CS, respectively.

The result which was presented in Fig. 3 implies that each module might contribute differently to the 43 diseases, while the statistical results of the chi-square (χ^2^) test (*p*-value < 0.001) further indicate the significant association of modules 1–8 to these diseases. The contribution propensity of each module to diseases was further discussed as follows.

It could be found from Fig. 3 that module 1, module 2, module 3, module 6, and module 8 commonly show a slight propensity toward diseases, but module 1 shows a notable contribution (*CS* = 1.000) to autism, compared with the other modules. In addition, it has been mentioned above that the targets in module 2 were connected by the “lysine degradation” pathway (hsa00310). Herein, the targets in module 2 might interfere with autism through lysine degradation, which has been already implicated in autism by previous studies^34–36^.

Compared to other modules, the targets in module 4, which were connected by the neuro-relevant “neuroactive ligand-receptor interaction” pathway (hsa04080), contribute to hypopituitarism (*CS* = 1.000) and mental disorders (*CS* = 0.667) most. Though hypopituitarism is a chronic endocrine illness, it is still regulated by the nervous system^37,38^. Thus, we inferred that the targets in module 4 would exert neuro-relevant function when affected by compounds.

The targets in module 5 were mainly connected by metabolism-relevant pathways, and they contribute to congenital developmental disorders (*CS* = 1.000), mental retardation (*CS* = 0.625), and inherited metabolic disease (*CS* = 0.503). Module 7 contributes to various diseases, including infectious disease (*CS* = 0.856), immune system disease (*CS* = 0.844), primary immunodeficiency (*CS* = 0.803), cancer (*CS* = 0.780), gastrointestinal disease (*CS* = 0.759), and autoimmune disease (*CS* = 0.750). Thus, it was inferred that the targets in module 7 would possess a higher possibility of exerting an immune-relevant function when affected by compounds, compared with other modules.

To ensure the reliability of the results, targets in the network that are associated with the drugs approved for the market are used to validate the association between diseases and network modules. The approved drug information and drug–target interactions were extracted from the Drugbank database^39^ and DrugCentral database^40^. The therapeutic area of the drugs was determined by the Anatomical Therapeutic Chemical Classification System code (ATC code), which is attributed to a drug by the WHO Collaborating Centre (WHOCC) for Drug Statistics Methodology^41^. The ATC code classifies drugs according to the following five levels: level 1, the organ or anatomical system on which they act; level 2, the pharmacological action; levels 3 and 4, the chemical, pharmacological, and therapeutic subgroups; and level 5, the specific single drug or drug combination. In this study, the ATC code of the approved drugs was abbreviated to level 1 to understand the organ or anatomical system on which they act.

For example, the ABL1 (tyrosine-protein kinase ABL1) has been reported to be targeted by the drug “dasatinib”, whose ATC code is L01XE06, and the connotation is shown as follows:

Level 1 (L): antineoplastic and immunomodulating agents

Level 2 (L01): antineoplastic agents

Level 3 (L01X): other antineoplastic agents

Level 4 (L01XE): protein kinase inhibitors

Level 5 (L01XE06): dasatinib

The level 1 abbreviation of the ATC code for dasatinib is L, which denotes that the therapeutic area of the drug dasatinib is antineoplastic and immunomodulating. Then, it was further inferred that the target ABL1 might be bound to exert antineoplastic and immunomodulating functions.

The verification result showed that, in the TPT network, 40 targets were identified to be targeted by approved drugs to exert therapeutic effects on the nervous system, among which 30 targets (~75%) are located in module 4, seven in module 5, and only two in module 6 and module 7, respectively. This indicates that the targets in module 4 contribute most to the nervous system, which is consistent with the contribution-scoring result; in total, 54 targets were identified to be targeted by approved drugs to exert antineoplastic and immunomodulating activity. Among these, 34 targets (~65%) are located in module 7, 15 (~25%) in module 5, two in module 1 and module 4, respectively, and only one in module 8. This indicates that the targets in module 7 contribute most to the immune system, which is consistent with the contribution-scoring result.

**Figure 4:**
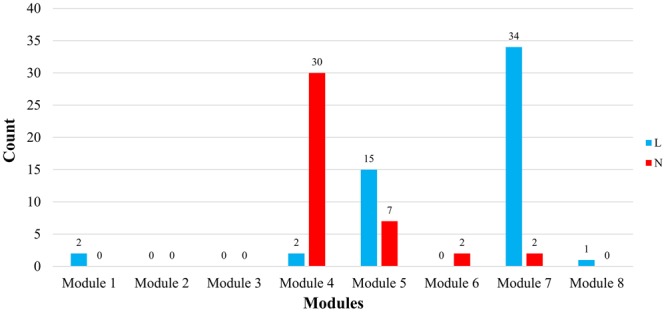
The count of approved drug targets in each module that are targeted to exert neuro-relevant function (N: Red) and antineoplastic or immunomodulating activity (L: Blue).

The verification results can also be found in Fig. 4. (Please refer to Supplementary Table S5 for detailed information of the targets with relevant approved drugs.) The chi-square (χ^2^) test (*p*-value < 0.001) of the results further indicates the significant association between network modules and diseases in terms of the targets of marketed drugs and the consistency in the main modules between predicted functions and drug indications in the real world.

### The module that contributes most to YPF-relevant diseases

**Figure 5:**
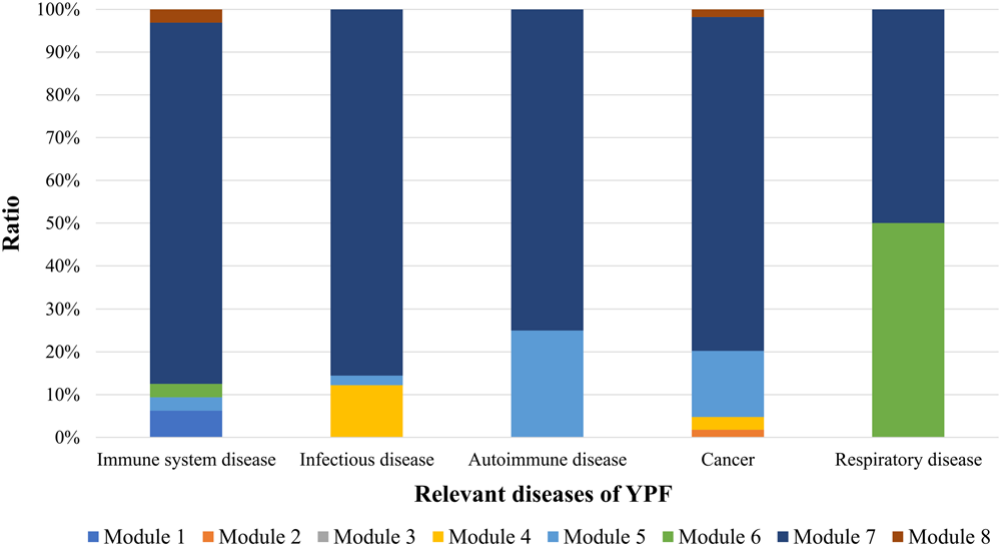
The contribution ratio of each module to relevant diseases of YPF decoction.

To understand which network module contributes most to the therapeutic effects of YPF decoction, the CS of each module toward relevant diseases of YPF was selected for the following analysis. The relevant diseases of YPF decoction are immune system disease^16,42–44^, autoimmune diseases^16,45,46^, infectious diseases^16,45,46^, respiratory diseases^20,46–48^, and cancer^49,50^, according to the information retrieved from the publications, which is listed in Table 2. (The term “relevant diseases” refers to the diseases that are affected by YPF, while the term “irrelevant diseases” denotes those diseases that are relevant to the enriched pathways of YPF targets but have not been reported to be affected by YPF). The ratio of the CS of modules 1–8 to relevant diseases of YPF is presented in Fig. 5, which indicates that targets in module 7 contribute most to immune system diseases, infectious diseases, autoimmune diseases, and cancer; respiratory diseases are mainly contributed to by targets in module 6 and module 7. From a general perspective, YPF decoction might interfere with the targets in module 7 with a higher possibility of exerting therapeutic activity.

**Table 2:** Reported diseases that are associated with YPF decoction.

Formula	Reported diseases	Disease category
YPF decoction	Allergic rhinitis, allergic airway inflammation, atopic dermatitis.	Immune system disease, autoimmune disease
	Respiratory tract infections, influenza, colitis, colonic inflammation, acute respiratory syndrome.	Infectious disease
	Pulmonary fibrosis, allergic asthma.	Respiratory diseases, autoimmune disease
	Chronic obstructive pulmonary disease.	Respiratory diseases
	Nasopharyngeal carcinoma, Lewis lung cancer.	Cancer

YPF decoction is a classic formula with immune-regulatory effects, and module 7 showed the highest potential for curing its relevant disorders compared to other modules. Thus, the 199 targets (nodes) were considered to be the potential therapeutic targets of YPF decoction. Please refer to Supplementary Table S6 for more detailed information about the targets.

Although YPF decoction is less known to exhibit anti-neoplastic activity directly in traditional applications, modern research has shown its potential in the treatment of idiopathic sweating of unknown cause in end-stage cancer patients^14^ and Lewis lung cancer *in vitro*^50^. There is extensive evidence showing a close relationship between inflammation and cancer^51–54^; that is, chronic inflammation contributes to cancer. Thus, the anti-neoplastic activity of YPF decoction might mainly occur via the regulation of the immune system.

### Identification of potential therapeutic targets in module 7

The above identification and CS of modules combined with statistical analysis were carried out to determine the therapeutic targets module that was highly associated with the therapeutic activity of YPF decoction. The following centrality analysis was conducted to evaluate the importance of the targets (nodes) within the therapeutic targets module from the perspective of the topological analysis of the TPT network. Four centrality indicators, i.e., degree centrality, closeness centrality, betweenness centrality, and eigenvector centrality, were used to measure the importance of the targets (nodes) from the different perspectives^55–57^. Then, an integrated indicator, namely target importance (*TI*), was calculated accordingly. Next, the top 10% therapeutic targets by *TI* were selected and recommended for further pharmacology study in the context of the mechanism illustration of YPF decoction. From a holistic perspective, YPF might interfere with targets in module 7 in a synergistic way, some of which (with higher centrality), as illustrated in Table 3, can be considered to be the most potential therapeutic targets of YPF. Moreover, the compounds relevant to these targets according to the SEA prediction can be recommended as potential active ingredients in YPF decoction in Table 3.

**Table 3:** Top 10% targets in module 7 ranking by *TI* and relevant compounds.

Target	UniProt ID	TI	Description	Relevant compound	Herb
PRKCA	P17252	1.000	Protein kinase C alpha type	Glycerol monolinoleate^§^	SR
				Glyceromonooleate^§^	SR
				Mandenol^§^	SR
MAPK3	P27361	0.940	Mitogen-activated protein kinase 3	Chrysin	AMR
PRKACA	P17612	0.834	cAMP-dependent protein kinase catalytic subunit alpha	Adenosine	AR, AMR
EGFR	P00533	0.685	Epidermal growth factor receptor	Quercetin^§^	AR
				Caffeic acid^§^	AR
				Tectochrysin^§^	AR
NFKB1	P19838	0.617	Nuclear factor NF-kappa-B p105 subunit	Psoralen^§^	SR
AKT1	P31749	0.612	RAC-alpha serine/threonine-protein kinase	Quercetin^§^	AR
GNAI3	P08754	0.610	Guanine nucleotide-binding protein G(k) subunit alpha	2-octanone	SR
CALM1	P0DP23	0.597	Calmodulin-1	Chrysin^§^	AMR
RAC1	P63000	0.592	Ras-related C3 botulinum toxin substrate 1	Guanosine	AR
ADCY5	O95622	0.584	Adenylate cyclase type 5	Adenosine	AR, AMR
RHOA	P61586	0.554	Transforming protein RhoA	Naringeninic acid	AR
CAMK2A	Q9UQM7	0.551	Calcium/calmodulin-dependent protein kinase type II subunit alpha	Ferulic acid	AR
NRAS	P01111	0.544	GTPase NRas	Ononin	AR
HRAS	P01112	0.544	GTPase HRas	Isoquercitrin	AR
HDAC1	Q13547	0.519	Histone deacetylase 1	Naringeninic acid^§^	AR
PLCG2	P16885	0.518	1-phosphatidylinositol 4,5-bisphosphate phosphodiesterase gamma-2	Glyceromonooleate	SR
CREB1	P16220	0.515	Cyclic AMP-responsive element-binding protein 1	Oroxylin A^§^	AR
				Chrysin^§^	AMR
				Wogonin^§^	SR
				Tectochrysin^§^	SR
GNAO1	P09471	0.512	Guanine nucleotide-binding protein G(o) subunit alpha	2-octanone	SR
CAMK2B	Q13554	0.511	Calcium/calmodulin-dependent protein kinase type II subunit beta	Isorhamnetin^§^	AR
				Quercetin^§^	AR
RXRA	P19793	0.504	Retinoic acid receptor RXR-alpha	Guanosine	AR

AR represents herbal medicine Astragali Radix (Huang qi in Chinese); AMR represents herbal medicine Atractylodis Macrocephalae Rhizoma (Bai zhu in Chinese); SR represents herbal medicine Saposhnikoviae Radix (Fang feng in Chinese).

^§^The compounds relevant to the particular targets with Tc values higher than the threshold value of SEA.

Previous studies have shown that YPF potentially attenuates allergic inflammation via the regulation of NF-kappa B activation^42^. Thus, it was inferred that YPF might also be relevant to NFKB1 according to the results of this study. Moreover, the top 10% potential therapeutic targets have been reported as a pleiotropic locus that is associated with cancer and a critical factor for acute inflammation associated with asthma, such as PRKCA^58,59^, EGFR^60^, AKT1^61^, RAC1^62,63^, RHAO^64,65^, HDAC1^66^. In the activation by GC, NR3C1 can activate MAPK8 (a seed gene) and several MAPK family members, including MAPK3, which could trigger GAB1 to interfere with asthma disease^67^; PRKACA shares a close relationship with cardiovascular disease and cancer^68^; ADCY5 has been found to play a critical role in sensory neuropeptide release and neurogenic inflammation and it is critical in the inflammatory process^69,70^; CAMK2A^71^ is a calcium signaling molecule involved in cell growth and stress-signals integration, and it has been reported to play a tumor-supportive role in osteosarcomas; PLCG2 is an enzyme responsible for ligand-mediated signaling in cells of the hematopoietic system, and it plays a key role in the regulation of immune responses^72^, CREB1^73,74^ and CAMK2B^75^ are also important for the regulation of immune responses; HRAS and NRAS, which belong to the p21 RAS subfamily of small GTPases, regulates cell proliferation, cytoskeletal organization, and other signaling networks, and they are among the most frequent targets of activating mutations activations in cancer^76^.

Moreover, six of these top-ranking targets have been reported to be targeted by approved drugs: EGFR, HDAC1, MAPK3, NFKB1, PRKCA, and RXRA; except for NFKB1, the others are targeted to exert antineoplastic or immunoregulating effects. The drugs cetuximab, gefitinib, afatinib, olmutinib, and brigatinib act on EGFR, and they have been used in cancer therapy; vorinostat and romidepsin act on HDA1, and they are used in primary cutaneous T-cell lymphoma; arsenic trioxide acts on MAPK3, and it has been used to treat acute promyelocytic leukemia; midostaurin acts on PRKCA to treat gastrointestinal stromal tumors; and RXRA is targeted by alitretinoin and bexarotene with the indication of Kaposi’s sarcoma skin lesions and primary cutaneous T-cell lymphoma, respectively.

All these previous studies provided validation for our novel TPT network-based study to some extent when using YPF as an example. As a result, targets with the highest centrality in this module are recommended as potential therapeutic targets of YPF decoction. Moreover, among them, there are eight most-important potential therapeutic targets, i.e., PRKCA, EGFR, NFKB1, AKT, CALM1, HDAC1, CREB1, and CAMK2B, which are identified by the relevance to YPF’s compounds with Tc value higher than the threshold of SEA method.

### The compounds associated with the top ranking targets

The compounds that were predicted to bind to the top-ranking targets (potential therapeutic targets) by SEA are presented in Table 3. Thus, these compounds were recommended as the potential active ingredients of YPF decoction. Among them, the compounds with a Tc value greater than the threshold of SEA are mainly flavonoids, including oroxylin A, psoralen, quercetin, tectochrysin, wogonin, chrysin, and isorhamnetin. This result is consistent with a previous report that the flavonoid in YPF shares immunomodulatory effects^9,42^.

## Methods

### Data acquisition and processing

YPF decoction is composed of Astragali Radix (*Huang qi* in Chinese, AR), Atractylodis Macrocephalae Rhizoma (*Bai zhu* in Chinese, AMR), and Saposhnikoviae Radix (*Fang feng* in Chinese, SR). First, the compound information of AR, AMR, and SR were collected from chemical databases, including the traditional Chinese medicines integrated database (TCMID, http://www.megabionet.org/tcmid/, which contains information from the TCM@Taiwan, traditional Chinese medicine information database (TCM-ID), herbal ingredients’ targets (HIT; accessed in May 2016) database, traditional Chinese medicine systems pharmacology (TCMSP; http://lsp.nwsuaf.edu.cn/tcmsp.php; accessed in May 2016) database, the cardiovascular disease herbal database (CVDHD, http://pkuxxj.pku.edu.cn/CVDHD/; accessed in May 2016), and the Chinese Academy of Sciences chemistry database (http://www.organchem.csdb.cn/scdb/; accessed in May 2016). Literature mining also supplemented the information from these databases. The common amino acids and compounds with high molecular weight, such as polysaccharides, were not included in this study. The names of compounds were standardised through SciFinder (https://scifinder.cas.org) according to the Chemical Abstracts Service (CAS) number. Additionally, these compounds’ PubChem CIDs were obtained from PubChem (https://pubchem.ncbi.nlm.nih.gov/) based on chemical names or structures.

The protein targets of compounds from YPF decoction were predicted by similarity ensemble approach (SEA: http://sea.bkslab.org/)^25^, which is a chemical similarity searching-based prediction method recognised worldwide for its accuracy^77,78^. Though the SEA method is capable of accounting only ~2,800 potential active proteins as alternative binding targets, the scale is consistent with the recent recognition of the druggable genome (~3000)^79–81^. In addition, the limitation of a chemical similarity searching-based prediction method, including the SEA, would be that the prediction is based on the two-dimensional (2D) structure (SMILE information) of compounds, which might produce the same potential targets of the isomeric compounds. Herein, it is not enough to predict solely using SEA or any other chemical similarity searching-based prediction method, when isomeric compound domains in the research. The NP-based study of YPF decoction involved 13 pairs of isomeric compounds in 352 compounds, which would interfere with the result slightly, from a holistic perspective.

Then, enrichment analysis is used to acquire target-pathway interactions which are essential for the further network construction and analysis. The targets were further enriched to KEGG pathways to create the targets–pathways relationship using the online tool STRING^82^ (http://string-db.org/). The enriched KEGG pathways with false rate discovery (FDR) < 0.01 were used in the subsequent research. The Kyoto Encyclopedia of Genes and Genomes^26^ (KEGG; http://www.genome.jp/kegg/pathway.html) was used to identify the diseases with which each pathway might be involved, the disease category information was used to represent the disease.

The diseases that are affected by YPF decoction were retrieved from previous research. First, publications about the topic of YPF were extracted from the Web of Science, and the topic keywords were set as “yupingfeng”, “Yu-Ping-Feng”, or “gyokuheifusan” (the Japanese name of YPF decoction). Second, the relevant diseases’ information was identified, where available. Then, the diseases were classified according to the KEGG database.

### Network construction and module identification

From a perspective of topology, the pathway-based associations between targets by enrichment analysis may be described as a network. A familiar representation is obtained by letting *N* be a set of nodes representing targets, and *E* be a set of edges where elements of *E* are unordered pairs of distinct nodes *n*_*i*_, *n*_*j*_ representing a pathway-based link between a target pair {*n*_*i*_, *n*_*j*_}. The two sets together are called a simple network *G* = (*N*, *E*). Specifically speaking, a TPT network was established on the one-mode targets–targets interaction, which was transferred by Pajek software^83^ from the two-mode “targets–pathways” relationship; then, the TPT network was visualised and analysed by Gephi software^84^. In the TPT network, the nodes refer to protein targets, and each set of two nodes is connected by an edge, which indicates that they are both involved in at least one of the same pathways.

Next, the Louvain algorithm for module identification incorporated in Gephi^32^ was adopted to explore the modularity structure of the TPT network. Since the targets in a module are connected by the same pathways or a functionally similar pathway, it was hypothesised that each module would exert a kind of specific function, and different modules would have effects on different diseases. Herein, an evaluation algorithm, named the contribution scoring algorithm, was established to weigh the contribution of each network module to various diseases and, further, to illustrate the TPT network.

### Contribution scoring algorithm

On the basis of an existing algorithm for measuring the associated intensity of individual elements between interactive sets^85,86^, an indicator measuring the intensity of associating a specific network module with a specific disease, named a “contribution score (CS)” was established in this study by integrating the modularity partition of the TPT network, the targets–pathways interactions, and the pathways–diseases interactions. In this algorithm, the higher the value (contribution score), the greater the contribution made by the investigated module for a particular disease. Then, the targets in the module with a significant high value toward relevant diseases affected by YPF were considered to be its potential therapeutic targets.

**Figure 6:**
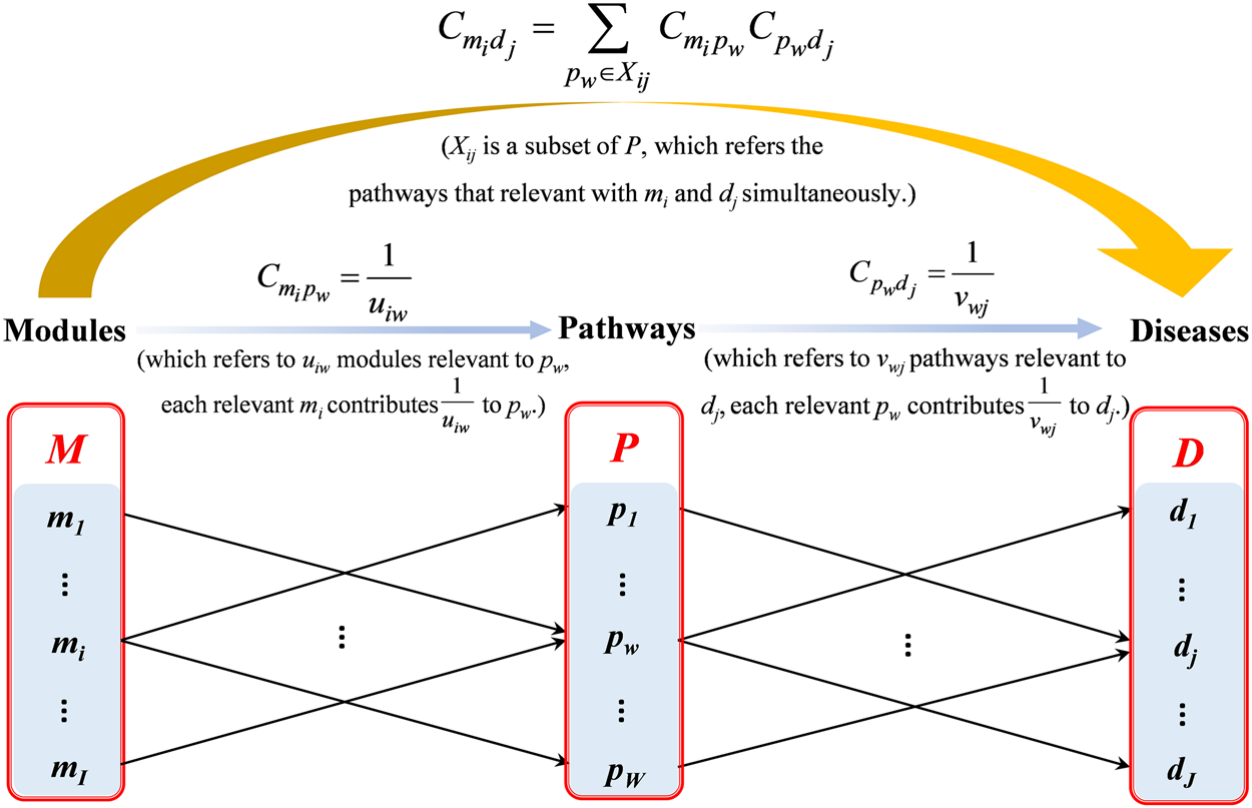
A diagram illustrating the contribution scoring algorithm.

Figure 6 presents a diagram for illustrating the contribution scoring algorithm, where *M* refers to a set of modules, *m*_*i*_ (*i* = 1, …, *I*); *P* refers to a set of pathways, *p*_*w*_ (*w* = 1, …, *W*); and *D* refers to a set of diseases, *d*_*j*_ (*j* = 1, …, *J*). In the TPT network, if *u*_*iw*_ modules are connected by *p*_*w*_, each relevant *m*_*i*_ contributes $\frac{1}{{u}_{iw}}$ to *p*_*w*_, namely the contribution of *m*_*i*_ to *p*_*w*_, ${C}_{{m}_{i}{p}_{w}}=\frac{1}{{u}_{iw}}$ ^85,86^. If *v*_*wj*_ pathways are relevant to *d*_*j*_, each relevant *p*_*w*_ contributes $\frac{1}{{v}_{wj}}$ to *d*_*j*_, namely the contribution of *p*_*w*_ to *d*_*j*_, ${C}_{{p}_{w}{d}_{j}}=\frac{1}{{v}_{wj}}$ ^85,86^.

The contribution scoring algorithm is defined as follows:

1 $${C}_{{m}_{i}{d}_{j}}=\sum _{{p}_{w}\in {X}_{ij}}{C}_{{m}_{i}{p}_{w}}{C}_{{p}_{w}{d}_{j}}$$

where *X*_*ij*_ is a subset of *P* and refers to the pathways that are relevant to *m*_*i*_ and *d*_*j*_ simultaneously, and where ${C}_{{m}_{i}{d}_{j}}$ refers to the CS of *m*_*i*_ to *d*_*j*_, which is the sum of the contribution of *m*_*i*_ to *d*_*j*_ through all its relevant *p*_*w*_ in *X*_*ij*_. The value of ${C}_{{m}_{i}{d}_{j}}$ varies from 0 to 1: the higher the value, the greater the contribution *m*_*i*_ might make to *d*_*j*_; and all the modules contribute 1 to a particular disease.

**Figure 7:**
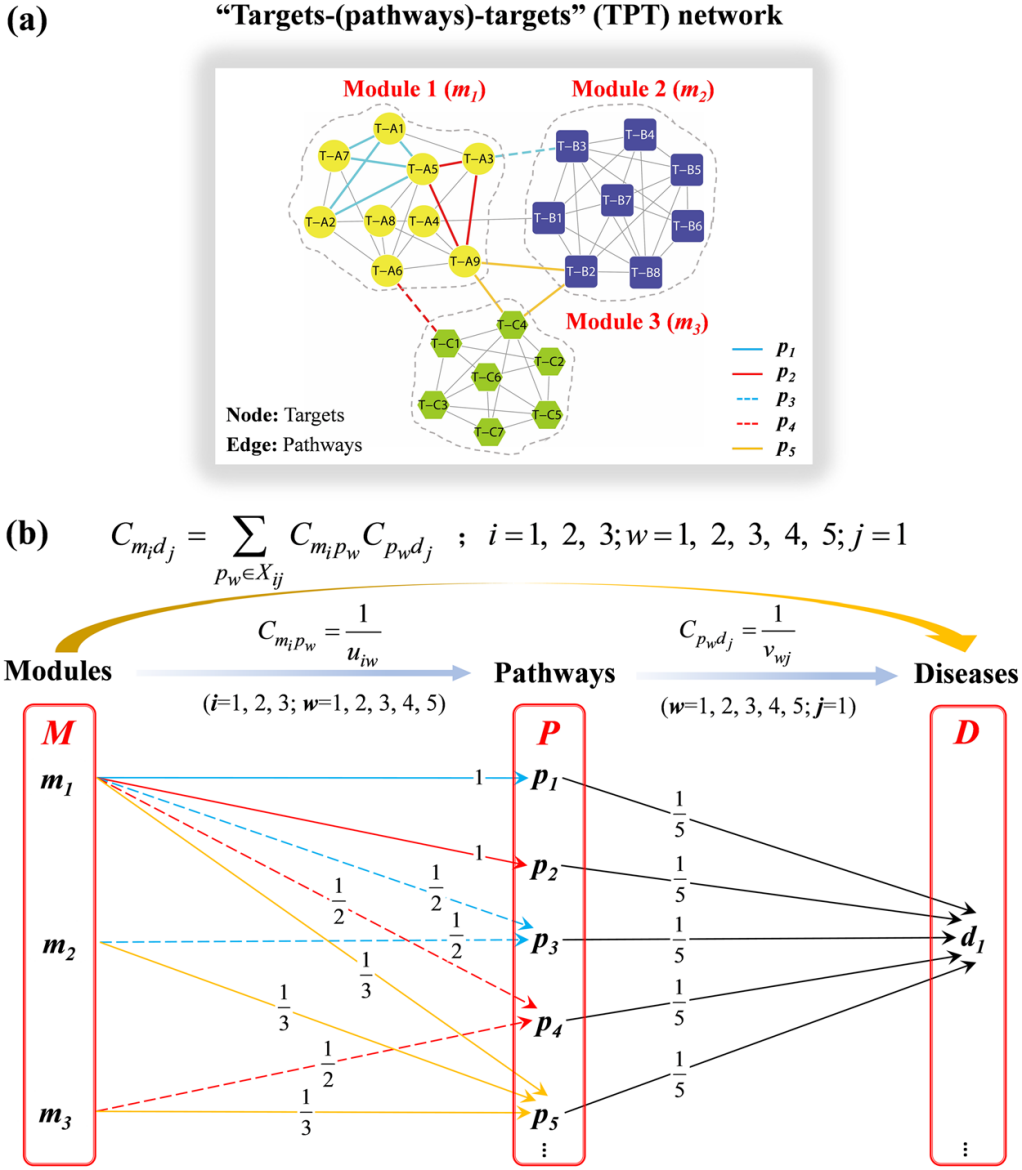
An example demonstrating the contribution scoring algorithm. (**a**) a TPT network with modules identified: In the diagram, the nodes represent targets, and the edges represent pathways. Nodes T-A1-T-A9 in yellow refer to targets in module 1 (*m*_*1*_); nodes T-B1–T-B8 in blue refer to targets in module 2 (*m*_2_); nodes T-C1-T-C7 in green refer to targets in module 3 (*m*_3_), assuming that *p*_1_ − *p*_5_ are pathways that are relevant to *d*_1_. (**b**) a pipeline abstracted from (**a**).

Below, an example is given to demonstrate the application of the contribution scoring algorithm. The Fig. 7(a) represents a TPT network with modules identified, and the Fig. 7(b) represents a pipeline which was abstracted from Fig. 7(a).

The TPT network was divided into three modules: module 1 (*m*_1_), module 2 (*m*_2_), and module 3 (*m*_3_). Since *p*_1_ and *p*_2_ connected the protein targets from only one module, *m*_1_, the contribution of *m*_1_ to *p*_1_ or *p*_2_ could be calculated as

2 $${C}_{{m}_{i}{p}_{w}}=\frac{1}{{u}_{iw}}=1,\,(i=1;\,w=1,2)$$

As *p*_3_ and *p*_4_ were contributed to by the protein targets from *m*_1_ and *m*_2_, and from *m*_2_ and *m*_3_, separately, the contribution of *m*_1_ or *m*_2_ to *m*_3,_ and from *m*_1_ or *m*_3_ to *m*_4_, could be calculated as

3 $${C}_{{m}_{i}{p}_{w}}=\frac{1}{{u}_{iw}}=\frac{1}{2},\,(i=1,2;\,w=3,\,OR\,i=1,3;\,w=4)$$

and since all three modules were related to *p*_5_, the contribution of *m*_1_, *m*_2_, or *m*_3_ to *p*_5_ could be calculated as

4 $${C}_{{m}_{i}{p}_{w}}=\frac{1}{{u}_{iw}}=\frac{1}{3},(i=1,2,3;w=5)$$

It was assumed that *d*_1_ was relevant to five pathways (*p*_1_ − *p*_5_) in the TPT network, herein, $${v}_{wj}=5,$$$$(w=1,\,2,\,3,\,4,\,5;\,j=1)$$, and that each pathway contributed $$\frac{1}{5}$$ to *d*_1_, namely:

5 $${C}_{{p}_{w}{d}_{j}}=\frac{1}{{v}_{wj}}=\frac{1}{5},(w=1,\,2,\,3,\,4,\,5;\,j=1)$$

Ultimately, according to equation (1), the result of

6 $${C}_{{m}_{1}{d}_{1}}=\sum _{{p}_{w}\in {X}_{11}}{C}_{{m}_{1}{p}_{w}}{C}_{{p}_{w}{d}_{1}}=\frac{2}{3},{X}_{11}=\{{p}_{1},{p}_{2},{p}_{3},{p}_{4},{p}_{5}\},$$

7 $${C}_{{m}_{2}{d}_{1}}=\sum _{{p}_{w}\in {X}_{21}}{C}_{{m}_{2}{p}_{w}}{C}_{{p}_{w}{d}_{1}}=\frac{1}{6},{X}_{21}=\{{p}_{3},{p}_{5}\},$$

8 $${C}_{{m}_{3}{d}_{1}}=\sum _{{p}_{w}\in {X}_{31}}{C}_{{m}_{3}{p}_{w}}{C}_{{p}_{w}{d}_{1}}=\frac{1}{6},{X}_{31}=\{{p}_{4},{p}_{5}\}$$

could be calculated: ${C}_{{m}_{1}{d}_{1}}$ shared the highest value, indicating that the targets in *m*_1_ contributed the most to *d*_1_ in the TPT network. That is, the targets in *m*_1_ might exert the most potential therapeutic proclivity toward *d*_1_ compared with the targets in other modules. Additionally, the total contribution of the three modules to *d*_1_ was 1.

### Statistical validation

The CS, which reflected the contribution of each target module to relevant diseases, could be calculated from the established contribution scoring algorithm. To validate the hypothesis that different modules would have effects on different diseases, the association between network modules and therapeutic diseases was examined by chi-square (χ^2^) test. The verification of the contribution scoring algorithm based on the information of targets and approved drugs was also conducted by chi-square (χ^2^) test.

### Centrality analysis

An integrated indicator, namely target importance (*TI*), was calculated accordingly as the equation below.

9 $$T{I}_{i}=\frac{1}{4}(\frac{D{C}_{i}}{ma{x}_{(DC)}}+\frac{B{C}_{i}}{ma{x}_{(BC)}}+\frac{C{C}_{i}}{ma{x}_{(CC)}}+\frac{E{C}_{i}}{ma{x}_{(EC)}})$$

where *TI*_*i*_ denotes the importance of *target i*, *DC*_*i*_, *BC*_*i*_, and *CC*_*i*_, and *EC*_*i*_ denotes the degree, betweenness, closeness, and eigenvector centrality of *target i*, respectively; meanwhile, *max*_(*DC*)_, *max*_(*BC*)_, *max*_(*CC*)_, and *max*_(*EC*)_ denote the maximum degree, betweenness, closeness, and eigenvector centrality in the therapeutic target module. The value of *TI*_*i*_ varied from 0 to 1, and the higher the value shared by a target, the more important the target was in the network from the topological perspective.

### Data availability

The source data for analysis in this work are available in PubChem [https://pubchem.ncbi.nlm.nih.gov/], SEA [http://sea.bkslab.org/], KEGG [http://www.genome.jp/kegg/pathway.html]. Drugbank [https://www.drugbank.ca/], Drug Central [http://drugcentral.org/]. The datasets generated during and/or analysed during the current study are available from the corresponding author on reasonable request.

## Conclusions

A novel NP-based method that could help discover the core mechanisms of herbal formulas from complicated information and interactive relationships was presented in this study. YPF decoction was investigated by using the NP-based method, which integrated the “multi-targets–multi-pathways–multi-diseases” relationship into a TPT network and a contribution scoring algorithm. The modules in the TPT network with a similar function, especially coinciding with the already known application of YPF decoction, were inferred from the finding that the protein targets in module 7 were highly associated with the underlying mechanism of YPF decoction, especially those with highest centrality as presented in Table 3. Additionally, some of these targets have been reported to be relevant to the therapeutic activity that YPF might exert. Herein, the network-based method provided an easier and more reliable strategy for uncovering the potential therapeutic targets of YPF.

In short, the proposed TPT network combined with the contribution scoring algorithm could be widely applied in the analysis of traditional medicines/formulas with robust effects in clinical care. And, importantly, the most therapeutically related modules coinciding with their clinical applications have a high chance of being associated with potential mechanisms worthy of further experimental investigation.

## Electronic supplementary material

Supplementary Table S1

Supplementary Table S2

Supplementary Table S3

Supplementary Table S4

Supplementary Table S5

Supplementary Table S6
